# Supplementary material for: Learning curve and surgical outcome of robotic assisted colorectal surgery with ERAS program
Source: Sci Rep. 2022 Nov 29;12:20566. doi: 10.1038/s41598-022-24665-w (PMC9709162; doi:10.1038/s41598-022-24665-w)
Supplement: Supplementary file 1 — Supplementary Information 1. [file 41598_2022_24665_MOESM1_ESM.zip › LSC Raw data-Ñ[▒K/140/O_ñw╝╨░O▒Kñσ.pdf]

正式摘要(電子簽章：000000227293070)

W83 / W83

臺中榮民總醫院

Operation Note

[redacted]

- 手術日期：2021/10/06
- 手術醫師：林俊余，R 林順鴻，R 林志安
- Anesthesia : GE
- Position : Supine

Diagnosis

- Pre-operative diagnosis : Malignant tumor of T-colon
- Post-operative diagnosis : T colon adenocarcinoma, cT1N0MO s/p right hemicolectomy on 2021/10/6

Surgical Indication

- To remove primary tumor

Operation

- 1. Laparoscopic right hemicolectomy with side-to-side anastomosis with EndoGIA 60mm*1 and handsewen technique
  2. Ileo-colic artery, right colic artery and middle colic artery was ligated via Hemo-clips.
  3. Take down of hepatic flexure : (+)
  4. Cut margin: proximal (ileum) >10 cm, (colon) 3 cm, distal 3 cm.
  5. Repair of mesentery defect : Nil.
  6. One 12 Fr. penrose drain was placed from RLQ area at Morrison's pouch.

Finding

- 1. No obvious lesion noted as pre-operatively, A clip was noted at T colon with tatoo. No enlarged lymph nodes at mesocolon.
  2. No other gross tumor was found.
  3. There was no visible liver metastasis or visible peritoneal seeding.
  4. Moderate adhesion between A-colon and adjacent small bowel, Some part of small bowel was moderately attached to the abdominal wall over the RLQ area

Procedure

- 1. General endotracheal anesthesia was induced and then the patient was placed in the supine position.
  2. Preoperative antibiotics were given. A Foley catheter and nasogastric tube were placed. The abdomen was prepped and draped in standard sterile fashion.
  3. A small vertical incision was made left to the umbilicus and a 12mm trocar was inserted into the peritoneal cavity by open method.
  4. Pneumoperitoneum was established with CO2 inflation. Laparoscope was inserted and then a 12mm trocar were inserted over LUQ of abdomen.
  5. Operative finding was mentioned above
  6. Another two 5mm trocar was inserted over LLQ, RLQ and RUQ of abdomen.
  7. Adhesion band lysis was performed carefully via electrical cautery and Ligasure scalpel.
  9. The small bowel was inspected and retracted to downward by grasper. The peritoneum overlying the mesentery was then scored with electrocautery and the ileocolic artery was identified, ligated with endoclips, and transected. Right colic artery and vein was also divided and ligated with hemoclip.
  10. After the vessel were all under controlled, the colon was freed from its peritoneal attachments along the line of Toldt from the cecum to the hepatic flexure.

- 11. Hemostasis was checked in the operative field. Cecum, ascending colon and hepatic flexure were mobilized and then laparoscope was removed.
  12. The midline incision was extended over left peri-umbilical area.
  13. The ascending colon was delivered into the incision and points of transection were selected proximally and distally.
  14. Due to moderate adhesion status over the terminal ileum area, after applying EndoGIA 60mm to resected the desired colon, the anastomosis was performed via side to side anastomosis with EndoGIA 60mm *1 and then COMMON CHANNEL was sutured with 4-0 monocryl with cornnel suture. The outer layer was strenghen with 3-0 mersilk
  16. The anastomosis was checked and found to be intact and widely patent.
  17. The small bowel and colon was put back into the abdomen. Pneumoperitoneum was established again.
  18. Massive distill water was administered to irrigated the anastmosis and the surrounding area to check and treat for any suspicious bleeders.
  19. One 12 Fr. penrose drain was placed at sub-hepatic region via RLQ trochar wound.
  20. The fascia was closed with interrupted sutures via Num.1 Vicryl. The fascia of the 12 mm trochar wound was closed with 1-0 Vicryl.
  21. The skin was sutured with interrupted method via 3-0 Nylon.
  22. The patient tolerated the procedure well and was brought to the POR in stable condition.
- Estimated blood loss: minimum
- 器械數量: 正確
- 紗布數量: 正確

Image

- PAINT
  - abdomen

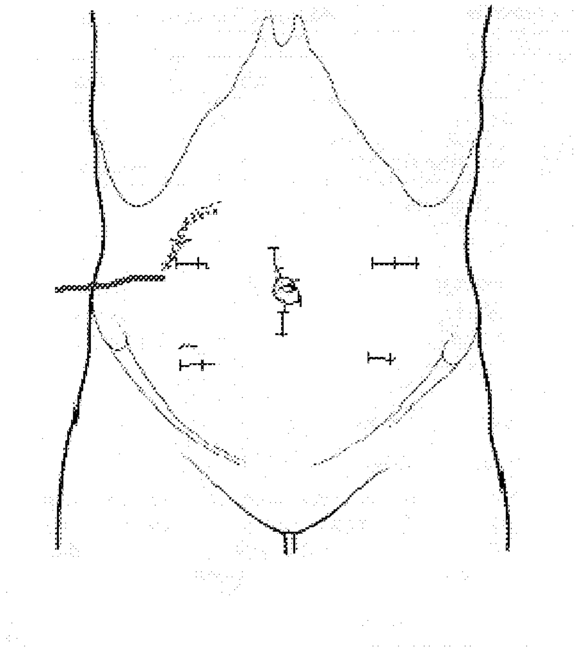

  - colon & rectum

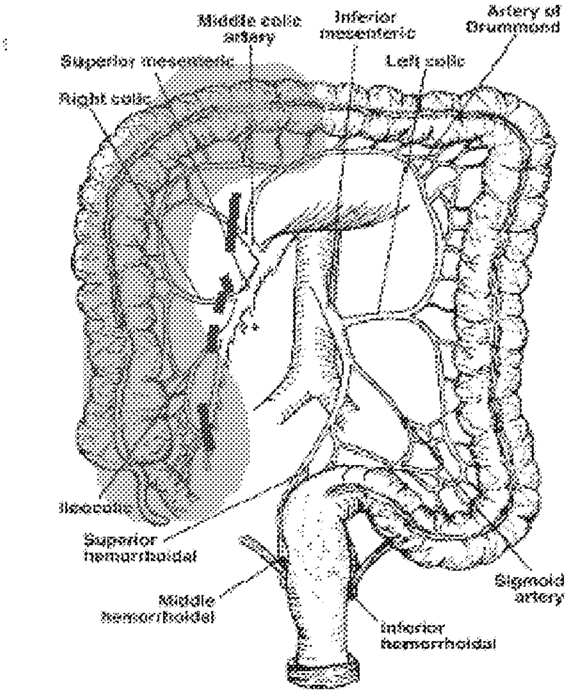

主治 2222H林俊余 / 醫師 2332G林志安

正式摘要(電子簽章：**000000227293070)**

W83 / W83

doctorRank:B
